# Accuracy of QuantiFERON-TB Gold Test for Tuberculosis Diagnosis in Children

**DOI:** 10.1371/journal.pone.0138952

**Published:** 2015-10-06

**Authors:** Michela Sali, Danilo Buonsenso, Delia Goletti, Pamela D’Alfonso, Antonella Zumbo, Giovanni Fadda, Maurizio Sanguinetti, Giovanni Delogu, Piero Valentini

**Affiliations:** 1 Institute of Microbiology, Università Cattolica del Sacro Cuore, Rome, Italy; 2 Institute of Pediatrics, Università Cattolica del Sacro Cuore, Rome, Italy; 3 Translational Research Unit, Department of Epidemiology and Preclinical Research, "Lazzaro Spallanzani" National Institute for Infectious Diseases (INMI), IRCCS, Rome, Italy; Chinese Academy of Medical Sciences and Peking Union Medical College, CHINA

## Abstract

**Objectives:**

To evaluate the accuracy of the QuantiFERON-TB Gold assay (QFT-IT) in children with suspected active or latent TB infection (LTBI).

**Methods:**

A retrospective study was conducted on 621 children (0–14 years old) evaluated for TB infection or disease. Following clinical assessment, children were tested with the QFT-IT assay.

**Results:**

Among the 140 active TB suspects, we identified 19 cases of active disease. The overall sensitivity for active TB was 87.5%, ranging from 62.5% in children 25–36 months old to 100% in children older than 49 months. The overall specificity for active TB was 93.6%. Among the 481 children tested for LTBI screening, 38 scored positive and all but 2 had at least one risk factor for TB infection. Among the 26 children with indeterminate results, bacterial, viral or fungal pneumonia were later diagnosed in 11 (42.3%) cases and non-TB related extra-pulmonary infections in 12 (46.1%).

**Conclusions:**

Our results indicate that the children's response to QFT-IT associates to active TB and risk factors for LTBI. Moreover, we show that mitogen response is also found in children of 1 year of age, providing support for QFT-IT use also in young children.

## Introduction

Children bear a substantial part of the tuberculosis (TB) epidemic at a global level. It is estimated that there were 530,000 childhood TB cases globally in 2013 [1], although it is problematic to obtain accurate data due to the many difficulties associated with TB diagnosis in children and the weakness of surveillance systems in countries where TB is endemic [2]. In children, the main risk factor for infection is exposure to adults with pulmonary TB, which, in children under the age of 2, is very likely to progress to active disease within the first year of primary infection [3]. Moreover, young children are more likely to develop the most severe forms of disseminated and meningeal TB, characterized by high mortality rate [4]. For these reasons, children under the age of 5 with no signs of TB disease but who have close contact with a contagious adult TB patient should receive isoniazid preventive therapy (IPT), which is known to drastically reduce the risk of developing active TB [4]. Ideally, IPT should only be administered to truly infected children, but the lack of effective diagnostic tools prevents this approach.

Diagnosis of childhood TB is notoriously challenging due to non-specific clinical and radiological signs and difficulty in obtaining a microbiological confirmation. TB disease is often diagnosed by a positive Tuberculin Skin Test (TST) result, epidemiologic information (exposure to a known source case) and a compatible clinical and radiographic presentation. For decades, the TST was the only test available for diagnosing latent TB infection (LTBI), though both false-negative and false-positive results plague this old assay [4].

More recently, interferon- release assays (IGRAs) have been developed to replace the TST for detecting LTBI, and while their intended use is not to diagnose active TB, some studies suggested their usefulness to identify childhood TB [5]. The sensitivity and specificity of the TST and IGRAs were shown to be similar for diagnosing active TB, while it remains difficult to determine their accuracy in detecting LTBI, given the lack of gold standards in children and adults [6] and the somehow controversial definition of LTBI [7,8]. In this regard, the Paediatric Tuberculosis Network European Trials group (PTBNET) has recently defined LTBI as ‘a positive immunological test result (either TST or IGRA) in the absence of active TB’[9]. As stated by the PTBNET, the proposed definition has a number of limitation, particularly in light of recent advances in TB pathogenesis. In fact, there is a growing understanding that the simple dichotomous classification of TB as “active” or “latent” is no longer acceptable and “TB infection” encompasses a wide spectrum of conditions, ranging from asymptomatic to lethal disease [10]. The lack of a clear edge between infection and disease is a major challenge for the clinician who face the problem to translate this new scenario in the routine clinical practice.

Although the TST has been widely used in clinical practice, it has known limitations, such as variable specificity, reproducibility, and cross-reactivity with non-tuberculous mycobacteria and BCG in those who have been vaccinated [11]. A major benefit of QuantiFERON-TB Gold-In-Tube (QFT-IT) (as well as other IGRAs) rests on its higher specificity in BCG-vaccinated subjects [12], preventing unnecessary and potentially toxic treatments [13–15]. However, the reliability of IGRAs in pediatric patients has been questioned. This is due to the lack of evidence of its performance in children under 5 years of age [16–18], (particularly in those under 2 years of age) mainly because of the reported high rate of indeterminate results and high variability among different studies. Indeed, even the recent NICE (National Institute for Health and Care Excellence) guidelines recommend the use of the TST rather than IGRAs for TB diagnosis in young children [19,20].

In this 4 -year retrospective cross-sectional study, we aimed to evaluate the accuracy of the QFT–IT among young children of different ages who were evaluated for LTBI screening and enrolled with suspected active TB.

## Methods

### Study Setting and Population

A retrospective cross-sectional study was conducted among children aged 0 to 14 years who were evaluated for TB infection or TB disease and referred to the Pediatric Infectious Disease Unit or outpatient clinic for the evaluation of internationally adopted children at the Catholic University of the Sacred Heart–A. Gemelli Hospital in Rome, Italy, during the period from January 2007 through July 2010.

The following category of children were enrolled in the study:

- Children with suspected active TB (symptomatic patients–children with suspected active TB)
- Children with known exposure to an active TB adult case (therefore children screened for LTBI);
- Clinically healthy, nationally or internationally adopted children evaluated by a national protocol for immigrants and nationally/internationally adopted children (see reference 12 for detailed list of screening tests performed), with or without known history of contact with adult active TB cases (therefore children screened for LTBI) approved by the National Working Group for Immigrant and Adopted Children, an official working-group of the Italian Society of Pediatrics and approved by the review board of our University.

Demographic and socio-economic characteristics, as well as complete personal and family histories in relation to TB exposure were recorded.

All patients were clinically evaluated and tested by QFT-IT (Cellestis Limited, Carnegie, Victoria, Australia). All children with a positive QFT-IT or high clinical suspicion of TB disease underwent radiological and microbiological investigations to confirm or rule out a diagnosis of active TB. Microbiological diagnosis included an acid-fast bacilli (AFB) examination following Ziehl–Neelsen staining; culture for *Mycobacterium tuberculosis* on Lowenstein-Jensen medium and in liquid media (BACTEC MGIT 960, Beckton-Dickinson, Maryland, USA); Strand Displacement Amplification (SDA) with PROBETEC ET^®^ (Beckton-Dickinson, Maryland, USA) for *M*. *tuberculosis*. Gastric washings were obtained on 3 consecutive days from all patients with suspected active TB. From those with suspected extra-pulmonary TB, samples were taken from different sites according to the suspected TB localization.

All children with a final diagnosis of active TB were evaluated for HIV infection using the combined serologic test Abbott Architect HIV COMBO–BioMérieux Vidas HIV DUO.

Diagnosis of LTBI was performed based on positive QFT-IT and normal chest radiography and in the absence of any clinical features that would suggest active disease [21]. Children who were diagnosed with LTBI were offered chemoprophylaxis with 6 months of isoniazid, in accordance with NICE guidelines [19].

Two definitions of *active* TB were accepted: *definite* (confirmed) and *probable* TB.

*Definite* TB was defined as the presence of at least one clinical specimen positive for *M*. *tuberculosis* on culture, positive acid-fast bacilli smear microscopy, or one histology sample positive for caseating granulomas or nucleic acid amplification test. *Probable* TB was defined as the presence of three or more of the following: 1) chest radiologic findings consistent with active TB; 2) typical symptoms such as fever, cough and weight loss; 3) other radiological evidence of active TB, including extra-pulmonary TB (e.g., computed tomography/magnetic resonance imaging findings consistent with TB meningitis) in addition to symptoms; 4) exposure to a case with active infectious TB; and 5) response to appropriate anti-TB therapy. We then classified sites of TB as pulmonary and extra-pulmonary TB. Children with active TB were treated according to the current NICE international guidelines [19].

Therefore, according to the definitions used, the following final diagnoses were performed:

- active TB
- LTBI
- Non TB children (children with no active TB neither LTBI); this group include both healthy/asymptomatic children without LTBI (Healthy) and all children with any diagnosis other than TB, bacterial, viral, fungal or parasitic infection (disease other than TB–dotTB)

Conventionally, we will define “foreign children” all children born out of Italy and children born in Italy by non-Italian parents. This is an internationally accepted classification since it has been clearly demonstrated that children born in TB endemic countries have a similar risk of contracting TB of children born in Italy from parents born in TB endemic countries, and both have a higher risk than children born in Italy from Italian parents [5].

### QFT-IT Assay

QFT-IT was performed as indicated by the manufacturer. Briefly, whole blood was collected in the QFT-IT tubes (Nil Control, TB-Ag and Mitogen) and incubated at 37°C for 16–24 hours. Following incubation, samples were centrifuged and the plasma was used to measure the IFN-γ produced in response to *M*. *tuberculosis* antigens, phytohaemagglutinin (PHA) and the negative control. Data were presented as IU/ml of IFN-γ; the cut-off value for a positive test was 0.35 IU/ml, according to manufacturer’s instructions.

### Statistical Analyses

Differences in frequencies were evaluated by the Fisher Exact Test. The median and IQR of IFN-γ production were calculated; the non-parametric Mann-Whitney U test was used to compare medians for unpaired comparisons and the Wilcoxon test for paired comparisons; the Kruskal-Wallis test was used to compare medians among the different groups. Data were analyzed using SPSS (SPSS, Chicago, IL) and Prism 5 software (Graphpad Software 5.0, San Diego, CA, USA). Differences were considered significant at p values ≤0.05.

### Ethical statement

This study was approved by the institutional review board of the Catholic University of Sacred Heart of Rome, Italy (prot 06/2015). The authors obtained verbal and written informed consent from the next of kin, caretakers, or guardians on behalf of the minors/children enrolled in the study.

## Results

### Study population

**Fig 1:**
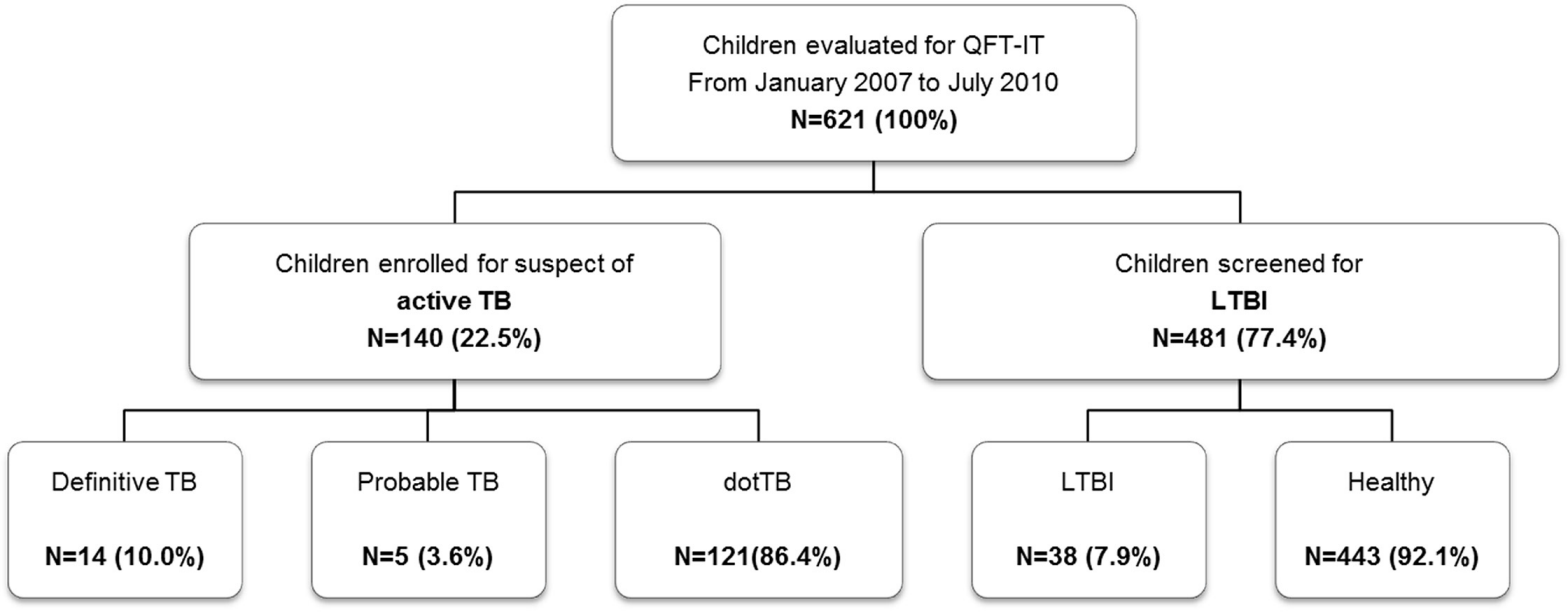
Flow chart of the study. (Abbreviations: TB: tuberculosis; LTBI: latent TB infection; QFT-IT: QUANTIferon TB Gold in tube.)

We evaluated 621 children, 275 (44.3%) were female; Fig 1 summarizes the children evaluated as patients with suspected active TB and children screened for LTBI. The mean age of the study population was 49 months (range 0 to 140 months) (Table 1). Regarding the origins, 180 children (28.9%) were Italian and 441 (71.0%) were foreigners, mostly born outside of Italy (394; 89.3%), and only 47 (10.6%) were born to foreign parents in Italy. All of the foreign children came from middle or high TB-burden countries (East Europe, Africa, South America, Asia) and most of them were internationally adopted children (352, 56.5% of the total study population).

**Table 1:** Demographic characteristics of the study population.

		Total N (%)	Suspect of active TB N (%)			LTBI screening N (%)		
		621 (100)	140 (22.5)			481 (77.4)		
		Total N (%)	active TB N (%)		dotTB N (%)	LTBI N (%)	Non TB N (%)	P value
			Definitive	Probable				
		621 (100)	14 (10.0)	5 (3.6)	121 (86.4)	38 (7.9)	443 (92.1)	
**Sex**	**Female**	275 (44.3)	7 (50)	1 (20.0)	54 (44.6)	17 (44.7)	196 (44.2)	0.844
**Age (months)**	**Median (IQR)**	49 (22–79)	16.5 (11.7–31.5)	29 (14–32.5)	40 (19–76)	67 (39.5–91.5)	51 (23–79)	0.001
**Classes of age**	**0–60 months**	378 (60.9)	13 (92.9)	5 (100)	85 (70.2)	16 (42.1)	259 (58.4)	0.0001
	**>60 months**	243 (39.1)	1 (7.1)	0	36 (29.7)	22 (59.4)	184 (41.4)	
**Origin**	**Italians**	180 (29)	2 (14.2)	1 (20.0)	77 (63.6)	7 (18.4)	95 (21.4)	0.0001
** **	**Foreign children**	441 (71)	12 (85.7)	4 (80.0)	44 (36.4)	31 (83.4)	348 (78.6)	

**Footnotes:** TB: tuberculosis; LTBI: latent tuberculosis infection; dotTb: disease other than TB

Overall, of the 621 children tested by the QFT-IT assay 59 (9.5%) scored positive, 536 (86.3%) scored negative and 26 (4.2%) were given an indeterminate result (Table 2). Among the 19 children with active TB, 17 resulted positive, 2 had an indeterminate result (with a lack of response to nil and mitogen) and none scored negative, providing a specificity of 100% for the QFT-IT.

**Table 2:** QuantiFERON TB Gold in tube (QFT-IT) results as a function of the diagnosis.

	Total N (%)	Suspect of active TB N (%)				LTBI screening N (%)		
	621 (100)	140 (22.5)				481 (77.4)		
		active TB N (%)		dotTB N (%)	p value	LTBI N (%)	Non TB N (%)	p value
		Definitive	Probable					
	621 (100)	14 (10.0)	5 (3.6)	121 (86.4)	0.0001	38 (7.9)*	443 (92.1)	N/A
**QTF responders N (%)**	59 (9.5)	13 (92.9)	4 (80.0)	4 (3.3)		38 (100)	0	
**QTF non responders N (%)**	536 (86.3)	0	0	96 (79.3)		0	440 (99.1)	
**QTF indeterminate N (%)**	26 (4.2)	1 (7.1)	1 (20.0)	21 (17.4)		0	3 (0.7)	

**Footnotes:** TB: tuberculosis; LTBI: latent tuberculosis infection; dotTb: disease other than TB

* by definition

### Children evaluated for active TB

Among the 140 children with suspected active TB, we identified 12 cases (57%) of pulmonary TB, 4 cases (19%) of extra-pulmonary TB (2 central nervous system, CNS TB, 2 lymph nodal TB); 5 cases (24%) of pulmonary and extra-pulmonary TB (4 cases of pulmonary and CNS TB, 1 case of pulmonary and chest wall TB). Microbiological confirmation was demonstrated in 14 (74%) children, while an alternative final diagnosis (dotTB) was made for 121.

### Children evaluated for LTBI

Among the 481 children tested for LTBI screening, 38 scored positive and all but 2 had at least one risk factor for TB infection (either known exposure with an adult index case, having been born in a TB-endemic country or having parents who were born in a TB-endemic country).

### Response to QFT-IT and age

**Fig 2:**
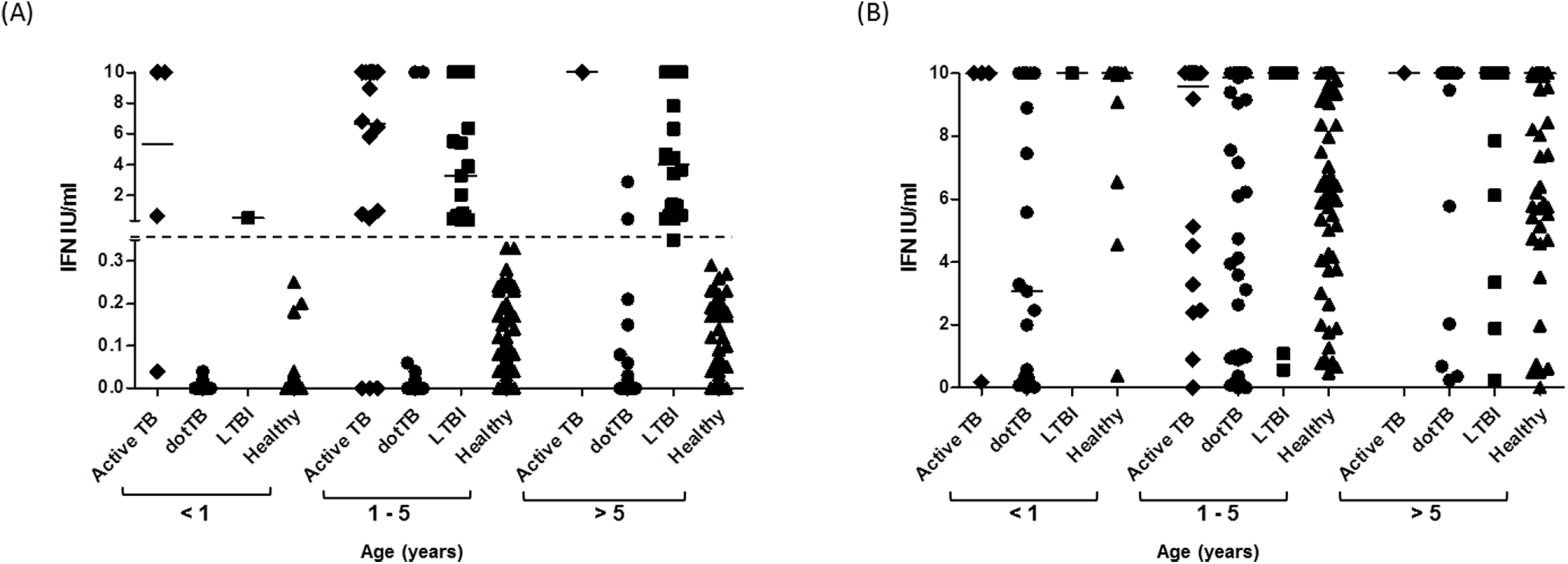
Quantitative response to QuantiFeron TB Gold-In Tube in relationship to age. IFN-γ levels in response to stimulation with *M*. *tuberculosis* antigens (A) and mitogen (B); individual QFT-IT results are plotted according to their final diagnosis and age. The cut-off value for a positive QFT-IT is represented by the *dotted line* at 0.35 IU/ml.

To investigate the relationship between age and ability to respond to the TB-specific antigens used in QFT-IT, we analyzed the IFN-γ response of all children showing a positive result (Table 3, Fig 2A). The proportion of positivity to QFT-IT ranged from 7.8% for the 25–36 months of age group to 10.9% for the 37–48 months of age group (Table 3), although the real significance of this value remains questionable given the non-homogeneity among the different age groups. Only 4 out of 52 children aged ≤ 12 months scored positive by QFT-IT; three of these children were diagnosed as active TB, while the fourth was a healthy child born in a TB-endemic country, diagnosed with LTBI. To note: one eight-month-old child with severe meningeal and cerebral TB (who did not survive) was scored QFT-IT indeterminate. In the 13–24 months age group, 11 out of 117 children scored positive and a final diagnosis of active TB was made for 7 of them. The remaining 4 children were diagnosed as LTBI: one child was a family contact of a smear-positive active TB adult, two children were born in TB-endemic countries, and for one child it was not possible to identify the risk factor for TB infection. In the 25–36 months of age group, six children scored QFT-IT positive and 5 of them were diagnosed as active TB. In the other three age groups (> 36 months), of the 38 children who scored positive by the QFT-IT, only two were diagnosed with active TB. Taken together, these results indicate that among the 21 QFT-IT -positive children ≤ 36 months, 15 were active TB and the remaining six were diagnosed as LTBI with a good correlation with TB risk factors.

**Table 3:** QuantiFERON TB Gold in tube (QFT-IT) results as a function of the age.

		Total N	Suspect of active TB N (%)			LTBI screening N (%)	
		621 (100)	140 (22.5)			481 (77.4)	
			active TB N (%)		dotTB N (%)	LTBI N (%)	Healthy N (%)
			Definitive	Probable			
age classes		621 (100)	14 (10.0)	5 (3.6)	121 (86.4)	38 (7.9)	443 (92.1)
**0–12**	**negative**	39 (75.0)	0	0	14 (11.6)	0	25 (5.6)
	**positive**	4 (7.7)	2 (14.2)	1 (20.0)	0	1 (2.7)	0
	**indeterminate**	9 (13.7)	1 (7.1)	0	7 (5.8)	0	1 (0.2)
**13–24**	**negative**	103 (88.0)	0	0	14 (11.6)	0	89 (20.0)
	**positive**	11 (9.4)	6 (42.9)	1 (20.0)	0	4 (10.5)	0
	**indeterminate**	3 (2.6)	0	0	3 (2.5)	0	0
**25–36**	**negative**	67 (87.0)	0	0	17 (14.0)	0	50 (11.3)
	**positive**	6 (7.8)	3 (21.4)	2 (40.0)	0	1 (2.7)	0
	**indeterminate**	4 (5.2)	0	1 (20.0)	3 (2.5)	0	0
**37–48**	**negative**	53 (82.8)	-	-	8 (6.7)	0	45 (10.7)
	**positive**	7 (10.9)	-	-	1 (0.8)	6 (16.2)	0
	**indeterminate**	4 (6.3)	-	-	3 (2.5)	0	1 (0.2)
**49–60**	**negative**	59 (86.8)	0	-	11 (9.1)	0	48 (10.8)
	**positive**	6 (8.8)	1 (7.1)	-	1 (0.8)	4 (10.8)	0
	**indeterminate**	3 (4.4)	0	-	3 (2.5)	0	0
**>60**	**negative**	215 (88.5)	0	-	32 (26.4)	0	183 (41.2)
	**positive**	25 (10.3)	1 (7.1)	-	2 (1.6)	22 (5.9)	0
	**indeterminate**	3 (1.2)	0	-	2 (1.6)	0	1 (0.2)

**Footnotes**: TB: tuberculosis; LTBI: latent tuberculosis infection; dotTb: Disease other than TB.

As shown in Fig 2A, all children with a positive QFT-IT test had an IFN-γ production in response to TB-specific antigens which was always > 0.53 IU/ml, while most of the negative results had IFN-γ values < 0.2 IU/ml.

### Mitogen and indeterminate results

It has been suggested that the immune system in newborns and infants may not be properly mature or capable of responding to antigenic stimuli *ex vivo* and for these reasons caution has been recommended for the use of the IGRAs in this population [22]. Measurement of the IFN-γ secretion in response to mitogen in IGRAs provides a useful indication of the potential ability of the immune system to respond to the antigenic stimuli. As shown in Fig 2B and Table 4, most of the children in the different age groups responded to the mitogen by secreting high levels of IFN-γ, indicating that the immune system is not impaired in its ability to mount an immune response in young children. Overall, the number of indeterminate results, which indicate subjects unable to properly respond to the mitogen, was low in the different groups with the only exception being the children enrolled for suspected active TB but diagnosed with a disease different from active TB (dotTB).

**Table 4:** Clinical characteristics of the 121 patients enrolled for active TB but with an alternative diagnosis (dotTB).

Pathology	Age in months												
	0–12		13–24		25–36		37–48		48–60		>60		TOT
N of patients	21		17		20		12		15		36		121
							N (%)						
Bacterial Pneumonia	10	(16.7)	8	(13.3)	9	(15.0)	7	(11.7)	10	(16.7)	16	(26.7)	60
Bacterial lynphadenitis	4	(28.6)	1	(7.1)	4	(28.6)	4	(28.6)	-		1	(7.1)	14
Fever of Uknown origin	1	(11.1)	4	(44.4)	1	(11.1)	-		1	(11.1)	2	(22.2)	9
Brain Tumor	1	(33.3)	-		1	(33.3)	-		-		1	(33.3)	3
Juvenile idiophatic arthritis	-		1	(33.3)	-		-		-		2	(66.7)	3
Seizure	-		2	(66.7)	1	(33.3)	-		-		-		3
Leukemia	-		1	(50.0)	-		-		1	(50.0)	-		2
Acute hepatitis	-		-		1	(50.0)	-		-		1	(50.0)	2
Achalasia	-		-		-		-		-		2	(100.0)	2
Growth failure	-		-		-		-		-		2	(100.0)	2
Constipation	1	(100.0)	-		-		-		-		-		1
Sepsis	1	(100.0)	-		-		-		-		-		1
Recurrent vomiting	1	(100.0)	-		-		-		-		-		1
Bacterial meningitis	1	(100.0)	-		-		-		-		-		1
AIDS*	1	(100.0)	-		-		-		-		-		1
Dermoid cyst	-		-		1	(100.0)	-		-		-		1
Intussusception	-		-		1	(100.0)	-		-		-		1
Chronic anemia	-		-		1	(100.0)	-		-		-		1
Erythema nodosum	-		-		-		1	(100.0)	-		-		1
Gastro-oesophageal reflux	-		-		-		-		1	(100.0)	-		1
Henoch Schonlein Purpura	-		-		-		-		1	(100.0)	-		1
Syphilis	-		-		-		-		1	(100.0)	-		1
Ostemyelitis b	-		-		-		-		-		1	(100.0)	1
Otomastoiditis	-		-		-		-		-		1	(100.0)	1
Urinary infection	-		-		-		-		-		1	(100.0)	1
Mononucleosis	-		-		-		-		-		1	(100.0)	1
Appendicitis	-		-		-		-		-		1	(100.0)	1
Recurent abdominal pain	-		-		-		-		-		1	(100.0)	1
Evans syndrome	-		-		-		-		-		1	(100.0)	1
Bacterial subdural abscess	-		-		-		-		-		1	(100.0)	1
Nephrosis	-		-		-		-		-		1	(100.0)	1

**Footnote:** AIDS: acquired immunedeficiency syndrome.

Among the 26 children with indeterminate results, bacterial, viral or fungal pneumonia or extra-polmonary infection were later diagnosed in 22 cases (80.8%), Table 5 reports the clinical features of all children scoring indeterminate. In Table 3, the group of children 0–12 months old showed a statistically significant higher rate of indeterminate results (9/52, 17.3%; p = 0.001) compared to the other age groups, with the higher proportion among the group classified as dotTB. Conversely, analyses of asymptomatic children screened for LTBI showed that in this group of 481 children, the rate of indeterminate results was particularly low (0.2%) and no statistically significant differences were found when the 0–12 months old group was compared with the older age groups.

**Table 5:** Clinical characteristics of the children scored indeterminate to QFT-IT.

Age (months)	Diagnosis	Group
0	Healthy newborn (6^th^ day of life)	Healthy
4	Intracerebral space-occupying process	dotTB
6	Pneumonia and Ongoing measles infection	dotTB
7	CMV pneumonia	dotTB
7	Acute respiratory failure in Congenital HIV infection	dotTB
8	Severe meningeal and cerebral TB—The child died because of CNS TB	active TB
8	Pneumonia	dotTB
8	Pneumonia	dotTB
10	Bacterial lymphadenitis	dotTB
14	Juvenile idiopathic arthritis	dotTB
16	Acute myeloid leukemia	dotTB
20	Fever of unknown origin	dotTB
29	Bacterial lymphadenitis in ongoing chickenpox	dotTB
32	Pulmonary TB (probable)	active TB
34	Candida pneumonia in ongoing Epstein Barr infection	dotTB
36	Pneumonia	dotTB
40	Respiratory distress	dotTB
42	Bacterial lymphadenitis	dotTB
42	Healthy child	Healthy
45	Bacterial lymphadenitis	dotTB
50	Pneumonia	dotTB
50	Pneumonia	dotTB
52	Pneumonia	dotTB
71	Growth failure	Non TB
111	Osteomyelitis	dotTB
128	Juvenile idiopathic arthritis	dotTB

**Footnotes:** CMV: cytomegalovirus; TB: tuberculosis; CNS: central nervous system; dotTB: disease other than TB.

To further investigate the possible correlation between inability to respond to mitogen and very young age, in Fig 2 we plotted the IFN-γ levels secreted in response to mitogen by children subdivided into 3 main age classes. Only 8 out of 22 (33%) children ≤ 8 months old were able to secrete ≥ 10 IU/ml of IFN-γ, while 9 (41%) children secreted less than 1.0 IU/ml of IFN-γ, indicating an impaired ability to respond to mitogen, even though no statistically significant difference was found (p = 0.23), probably due to the low number of evaluated children under 12 months old. Conversely, children older than 8 months responded properly to the mitogen. There was no statistically significant effect of age on the magnitude of the response (p>0.05).

### Follow-up

At follow-up (min. 3 years, max. 6 years), none of the evaluated children who were not initially diagnosed with a disease other than TB (dotTB) received a later diagnosis of active TB.

## Discussion

Diagnosis of TB in children is a challenging task and the reliability of the new IGRAs, which are being effectively used in the adult population, awaits confirmation, primarily in those under 5 years of age. Here, we report the results of a retrospective study carried out on 621 children and we show that young children older than 1 year are capable of properly responding to the mitogenic stimuli and RD1 antigens when infected with *M*. *tuberculosis*.

In this study, the overall sensitivity of QFT-IT in children with active TB was 87.5%, ranging from 62.5% in children 25–36 months old to 100% in children older than 49 months, in line with previous reports [5,23] and with recent data from a multicenter study in Italy that included only < 2 years old children, where a sensitivity of 92,4% for QFT-IT was registered [24]. Conversely, Chiappini E. et al [25] measured a sensitivity of 73,3% for QFT-IT for active TB in < 5 years old children, which was much lower than what detected in parallel with TST (90,0%). These results highlight the need for more robust results on the use of IGRAs for the diagnosis of active TB, though the data of this study indicate a high sensitivity of QFT-IT. We also observed a high overall (93.6%) and age-group (> 90%) specificity of QFT-IT for active TB, similarly to what recently observed in other studies [24,25].

The lack of a gold standard for LTBI prevents proper assessment of QFT-IT performance for this group. It is remarkable that 36 out of 38 children (95%) scoring positive to QFT-IT, and therefore diagnosed with LTBI, had at least one risk factor for TB infection (either known exposure with an adult TB index case, being born in a TB-endemic country or having parents born in a TB-endemic country).

A major concern in the use of IGRA assays in young children is that the potentially immature immune system may result in an impaired capacity to secrete IFN-γ in response to antigenic stimuli. The determination of IFN-γ in response to mitogen in the QFT-IT assay allows for immune system status assessment and identifying subjects with impaired immunity who result indeterminate. In this study, only 26 children (4.2%) scored indeterminate with the rate being lowest in children older than 12 months of age (ranging from 6.25% in children aged 37–48 months to 1.2% in children older than 60 months). Conversely, we reported a 17% of indeterminate results in the 0–12 month-old-children, which is in line with previous data [15,22,26,27]. Recently, Critselis et al. found that QFT-IT indeterminate results occurred more frequently among children under 2 years of age (4%) than children older than 4 (1.7%) [28]. It has been suggested that subject age may be the main reason for the high level of indeterminate results, although a detailed analysis of the health status of these infants (as provided here) was lacking. In our study, only 2 out of the 26 young children with indeterminate results were asymptomatic children, and one of these two was a 6 -day- old newborn. Two of the children with indeterminate QFT-IT were diagnosed with active TB (one with a severe CNS TB that progressed to exitus) and 11 children were diagnosed with pneumonia.

Among the children who scored indeterminate, Epstein Barr Virus (EBV), Varicella Zoster Virus (VZV) or measles infections were identified in 4 out of the 26 children in our series, supporting the findings that ongoing viral infections may be common causes of indeterminate results [22]. Interestingly, 3 children with bacterial lymphadenitis undergoing amoxicillin-clavulanate therapy had indeterminate results, probably because of the ability of β-lactam benzylpenicillin to bind IFN-γ and reduce its availability for detection in the immune assays [29,30]. Moreover, 2 children with indeterminate results had juvenile idiopathic arthritis, a well-known cause of a negative TST [31].

Hence, with only one exception, all children with indeterminate scores had a reasonable explanation for the inability to respond to the mitogen, indicating that young age alone (being under 2 years of age, as suggested by the majority of studies) should not be considered the only cause of an indeterminate result, in line with recent findings by Debord et al. [32]. Acute infections seem to be the cause of the high proportion of indeterminate results and for this reason we evaluated the incidence of indeterminate results when an acute infection other than TB was diagnosed.

The performance of the QFT-IT in children living in high burden settings raised some concerns, as highlighted by a study where 27% of the children tested showed an indeterminate result, with higher rates among the under 2-year-old population and with no statistically significant association with HIV infection, sex or malnutrition [33]. Moreover, an indeterminate QFT-IT result at baseline was associated with subsequent increased childhood mortality [33] in line with previous data [34] and our experience where the only child who died for meningeal TB disease scored indeterminate for the QFT-IT. Although the high rate of indeterminate QFT-IT results in the pediatric population has always been considered an important limitation of this test, the possibility of having a third option in addition to “positive” and “negative” is an important, perhaps underrated advantage of the QFT-IT over the TST. In fact, when there is a high clinical suspicion of TB disease, an indeterminate QFT-IT result could be read as a red flag indicating an important cause of immune suppression or active severe TB requiring urgent and extensive diagnosis [33,34]. In this context, the results of this study further highlight the need of a non-adaptive immunity based diagnostic for children in TB endemic countries.

Our study has some potential limitations. First, the TST was performed in less than half of the patients, mainly because of the difficulty the parents had to make a return visit with the children. Hence, we decided not to include TST results in the analyses and to focus on QFT-IT. Second, BCG vaccination was registered in about 40% of the subjects, mainly due to the lack of valid certification in foreign-born children, thus, we did not include BCG vaccination in the analysis. However, it is well established that BCG vaccination does not impact QFT-IT status. Additional research is needed to improve current immune diagnostic tests for TB, including the use of new *M*. *tuberculosis* antigens different from those used in the QFT-IT [35–37], new immunological markers beside IFN-γ [38] and/or read-outs different from ELISA [39].

In conclusion, the present study indicates that even young children are able to respond to specific *M*. *tuberculosis* antigens and mitogen, providing support for using the QFT-IT assay also in young children, particularly those evaluated for LTBI.
